# The Role of Sex in Intimate Relationships: An Exploration Based on Martin Buber’s Intersubjective Theory

**DOI:** 10.3389/fpsyg.2022.850278

**Published:** 2022-04-25

**Authors:** Wei Zhang

**Affiliations:** Department of Psychology, Institute of Education, China University of Geosciences, Wuhan, China

**Keywords:** sex, intimate relationship, intersubjective interaction, I-It relationship, I-Thou relationship, psychoanalysis, attachment

## Abstract

On the basis of Buber’s distinction between “I-It” and “I-Thou” relationships, this paper explores the role of sex in intimate relationships by analyzing research in the fields of psychoanalysis and attachment theory. In the “I-Thou” relationship mode, both parties are often able to fully participate in the current sexual behavior and respond wholeheartedly. When there is incoordination (or even conflict) in sexual activities, they can negotiate sincerely, and can even repair the relationship if it breaks down. In the “I-It” relationship mode, sex exists more as a tool to achieve a certain purpose (e.g., economic guarantee, sense of security and sense of control), and the intersubjective relatedness is abnormal: either the boundary will be blurred and others become my vassal (control strategy) or I become others’ vassal (compliance strategy); or the relatedness will be cut off, leading to loneliness or false independence (avoidance strategy).

## Introduction

*It is a oneness that transcends all else. I feel overwhelmed and in a grand passion, and very close to the person I love.*

—a female interviewee (Hite, 1981a, p.425).

*The warmth, the sight, smell, sound, feel, and taste of her body is exquisite. I’m sure I sound like a broken record to her, but every one of my senses all over transmits wonderful feelings to me when I’m inside her.*

—a male interviewee (Hite, 1981b, p.325).

Sex is not so important in contemporary psychoanalysis as it was in the early stages of the field. This decreasing emphasis can be seen in the significant slowing of the publication of relevant literature (Fonagy, 2008; Shalev and Yerushalmi, 2009). Nevertheless, as a force closely related to reproduction and progeny, the influence of sex on human beings cannot be ignored. In the relational network that shapes individuals, intimate relationships are closely related to sex. Some analysts even claim that sexuality dominates (Mitchell, 1988). From a relational or intersubjective perspective, sexual behavior is an important way for partners to communicate and interact. As de Vries (2009) said, “When we’re in love, sex is one (very intimate) way of communicating with the other, of expressing our feelings. Sex is a body language in which we can express gentleness and affection, anger and resentment, superiority and dependence far more succinctly than we can verbally” (pp. 25–26). Therefore, the exploration of the role of sex in intimate relationships remains a valuable research theme.

As a description of human relationships, Martin Buber’s intersubjective theory is enlightening and has influenced many psychoanalytic researchers. However, compared with its influence on humanistic psychology, the application of Buber’s theory to psychoanalysis continues to call for further study (Orange, 2010). In his view, there are two basic modes in intersubjective relationship: “I-It” and “I-Thou.” In the former mode, I regard others as cognitive objects or tools to be manipulated; this stance will degrade the subjective status of others and also lead to my own alienation. In the latter mode, others and I are equal subjects. We devote ourselves to the current dialog and greet each other with a sincere attitude (Buber, 1970). In this study, we combine the research of psychoanalysis and attachment theory, with a focus on heterosexual relationships, to explore interactions in intimate relationships with the “I-It” and the “I-Thou” modes. Through this analysis, we can see that the role of sex in intimate relationships is constantly changing.

## Sexual Relationships Dominated by the “I-Thou” Mode

According to Buber, in an “I-Thou” relationship, the other is treated as a relatively independent subject. This means that the subject is able to understand differences with others (e.g., others have their own feelings, ideas, needs and abilities), and participants in such a relationship interact with each other with sincerity (Morgan and Guilherme, 2013). In the sexual behavior aspect of intimate relationships, “the eros of dialogue means the turning of the lover to the beloved in his otherness, his independence, his self-reality, and with all the power of intention of his own heart” (Friedman, 2002, p. 88). To sum up, this mode mainly involves the following two characteristics: (1) both partners fully participate in the current sexual activities and respond wholeheartedly; (2) when the sexual activities are uncoordinated (or even conflict), both partners can negotiate with sincerity, and the relationship can even be repaired after a breakdown.

### The Present Wholehearted Response

In sexual behavior patterns dominated by the “I-Thou” relationship, we can see an ideal situation – the perfect combination of emotion and sexual desire. Johnson (2008) referred to it as *synchrony sex*. In this type of sex mode, the touch at the physical level and the opening and response at the emotional level can achieve coordination, bringing both sides into a self-evident and deep sense of fusion. Physically, the contact between skin is so close, and the switching between touch and being touched is so frequent, that the body boundary between individual and others becomes very blurred and even seems to be erased (Stein, 2008; Kernberg, 2011a). At this point, many men and women describe a sense of “boundary ablation” during orgasm, such as becoming one, merging as one, oneness, and togetherness (Hite, 1981a,b). Similarly, Josselson (1992) said, “In sharing orgasm, we can, for a moment, be united in a state of oceanic boundarylessness—at one” (p. 74).

Emotionally, the opening and response of affect is so smooth that it is comparable to the “mind-to-mind” communication in mother-infant interactions, so that the emotional experiences of both sides are closely intertwined. Thus, we can see many aspects of emotional communication and conjunction in this kind of sexual intercourse, such as love, intimacy, closeness, warmth, acceptance, sincerity, safety, and satisfaction (Hite, 1981a,b). Johnson (2008) drew an analogy with the situation of a couple who had been married for many years when they danced the Argentine Tango together: “They are completely present and engaged with each other. Their moves are achingly deliberate, totally playful, and stunningly erotic. They are so attuned and responsive to each other that even though the dance is fluid, improvised in the moment, they never miss a step or a turn. They move as one, with grace and flair.”

It is worth noting that although the experience of “intimacy” is very strong, the subjectivity and differences between the two sides have not been completely erased. If we regard the sameness/difference between the individual and others as a continuum, then in the pole of sameness, the individual and others are exactly the same, extending each other, haunted by the sense of familiarity, but also obliterating the particularity of both sides; in the pole of difference, the individual and others are completely different, distinguished from each other, the subjectivity and uniqueness are highlighted, but also isolated by the sense of strangeness. Undoubtedly, the state of “strong union” and “togetherness” in sexual intercourse is very close to the pole of sameness, but the difference has not been completely eliminated. At this point, it can be seen that many men and women describe the blissful experience of orgasm by referring to certain body parts (e.g., penis, vagina, chest and face), as well as body movements (e.g., hugging, twitching and breathing) of each other (Hite, 1981a,b). This means that they are still able to perceive and distinguish the different bodies of both sides at certain times. In Fonagy (2008) words, “Each partner is, momentarily, both alone and fused with the other” (p. 26). In the view of many analysts, sexual desire inevitably has a dimension of “otherness”: in both real and fantastic sexual intercourse, individuals need to transcend and arouse their own psychological state in others, in order to achieve sexual pleasure (e.g., Mitchell, 2002; Stein, 2008; Kernberg, 2011b). Therefore, mature sexual excitement must be experienced in the other (Fonagy, 2008). In this sense, synchrony sex needs to be completed in cooperation with a similar but different person. It can be said that this stems from a process similar to the “mutual finding” described by Togashi (2012), i.e., both sides of the interaction find oneself and not-oneself in each other. Only by maintaining a dialectical tension of sameness and difference can we not only enjoy the pleasure of sex, but also not eliminate the subjectivity of each other. In the words of Mitchell (2002), it is a balance between safety and adventure.

This undigested “difference” is especially obvious in “solitude after intercourse.” According to Winnicott (1965), if after having satisfying sex, every partner can feel satisfied in solitude, it means a healthy relationship. In Fromm (1956), it was seen as a form of mature love, i.e., “union under the condition of preserving one’s integrity” (p. 16). Today, many analysts continue this view (e.g., Clulow, 2009) and regard this ability to be alone as an essential foundation for enjoying sex. In short, this kind of solitude can be regarded as a balance between relatedness and individuation; that is, individuals can not only establish a good relationship with each other, but also maintain their own autonomy.

In this kind of synchrony sex, sex becomes “a safe adventure.” As a result, sexual coordination and emotional stability complement each other. Specifically, it brings the following positive effects in the unconscious and conscious field. First, because both sides can immerse themselves in it and get full sexual enjoyment, it can strengthen the emotional connection and bond, and make both sides feel closer (Johnson, 2008). According to a national survey report, sexually satisfied partners are more likely to show their desire for partners, feel their desire for partners, reach orgasm in their sexual life, and have a higher level of emotional intimacy and hug outside of their sexual life (Frederick et al., 2017). Second, with a strong emotional connection, the confidence of both sides is further enhanced to make communication more smooth. In this regard, both parties are not only more able to frankly reveal their own shortcomings and desires in sexual behavior, without fear of being rejected by the other party, but they are also more able to speak frankly about their preferences or taboos in sex, without feeling embarrassed or offending the other party (Johnson, 2008). However, in this kind of relationship, sexual skills are often only an auxiliary rather than a main purpose (Fromm, 1956). Of course, both sides do not exclude new attempts: “Lovers can be tender and playful one moment, fiery and erotic another. They can focus on achieving orgasms in one interlude and in the next on gently journeying to the place poet Leonard Cohen calls ‘a thousand kisses deep”’(Johnson, 2008). Third, damaged internal and external relationships can be repaired, even the deficiencies brought by earlier relationships (Blechner, 2006). In the words of Scharff (1982), “good enough sex” updates the old relational pattern.

### Negotiation and Repair

Clearly, perfect cooperation in sexual behavior represents an idealized situation. In actual intimate interactions, some lack of coordination, out of step actions, interruption and even conflict are inevitable. In fact, there are many differences in sexual behavior between men and women. For example, studies have shown that men tend to have more sexual desire than women, specifically as follows: (1) men masturbate more frequently; (2) men desire sex more frequently and are more likely to feel unsatisfied; (3) in the process of intimacy promotion, men tend to be the party who wants to have sex earlier; (4) men have higher frequency of sexual fantasizing; (5) men are more willing to engage in sexual consumption, such as buying sex toys and pornographic materials; and (6) men are more willing to accept casual sexual behavior (Miller, 2015). In addition, a series of factors, such as age, sexual preference, physical health and the old object relations (or attachment style), also affect the degree of “sexual matching” between the two sides and then affect the sexual activities of the partner. In these “disharmonious” circumstances, negotiation and repair are particularly important.

In sexual activities dominated by the “I-Thou” relationship, even if there is an uncoordinated situation, both sides can often have good and effective sexual communication. For example, in the first story in Chapter 9 reported by Hite (1981b), a man initially knew nothing about women’s masturbation and thought that sexual intercourse was the only correct way for women to achieve orgasm. Later, after good communication, the man began to help a woman masturbate, and they began to consistently explore each other’s sexual preferences. With more familiarity with these preferences, both sides reached agreement and coordination in sexual life, and their sexual life ultimately became satisfactory. More importantly, the relationship between the two sides became very close.

Similarly, in another story of remarried women reported by Hite (1981a), when a woman confessed that she would masturbate, she felt very ashamed. However, the man accepted this, and repeatedly said that it was a normal thing, and that the woman’s appearance was very attractive. As a result, after the sex life blossomed, both sides went forward hand-in-hand and had a high degree of sexual satisfaction, and their married life was also very sweet. Many follow-up studies also support this point: individuals higher in sexual growth beliefs (i.e., believing that sexual satisfaction comes from hard work and effort) are more satisfied with their relationships, while those high in sexual destiny beliefs (i.e., believing that sexual satisfaction comes from finding a compatible sexual partner) experience lower relationship satisfaction when facing sexual differences (Maxwell et al., 2017). In terms of attachment style, whether homosexual or heterosexual, secure attachment people tend to have a good way of sexual communication, they are more interested and open to exploring sexual interactions with their partners, and they thus have the most satisfying sexual lives (Birnbaum, 2010; Mikulincer and Shaver, 2013).

In some difficult situations, the sexual relationship may break down; at this time, repair is particularly important. For instance, one of the partners may have a physical barrier that leads to the inability to have sex or the disharmony of the sexual relationship. Generally speaking, if both partners can enhance communication, improve some skills, overcome or solve physical problems, they can successfully tide over the “crisis” without seriously threatening the relationship (Scharff, 1982). In more serious cases, sexual disorders can endanger the entire family relationship. For example, in a case reported by Scharff and Scharff (1991), Lars (husband) was deeply troubled by premature ejaculation, while Velia (wife) said that she did not like and hated sex. Their sexual lives were negative, and this tense relationship also affected the whole family. However, when such partners are determined to face the problem, constantly communicate and explore treatment, and strive to overcome the difficulties together, their emotional connection becomes stronger. In the end, both sides get a high degree of satisfaction with the sex life, and the family relationship becomes more harmonious. In this sense, it is similar to “rupture and repair” described by Beebe and Lachmann (2002); that is, repair has changed both sides’ expectations of difficulties: sexual barriers are not “fatal” and not invincible. The partners will finally see the “sunshine when the rain is over” (Wallin, 2007). This further increases the confidence of both sides to move forward hand-in-hand and to jointly cope with difficulties.

In fact, compared with “transient” sexual excitement, “lasting” emotional intimacy is a glue that sustains intimate relationships for a longer time and tends to allow relationships of the “I-Thou” mode to be healthier. Research shows that there is a stage of retreat of passion in intimate relationship (Frederick et al., 2017). The beginnings of many intimate relationships are full of freshness and excitement when both men and women are enthusiastic and sexually active in exploring each other’s bodies. In addition, with the “embellishment” of idealization, both parties may be immersed in the passion of romantic love (Mitchell, 2002). However, the “excited period” will soon end and transition into the “dull period” (de Vries, 2009). At this time, the importance of emotional intimacy is particularly prominent. In Hite (1981a,b) report, many men and women said that although orgasm is attractive, it is not essential; more important is the level of intimacy felt by both partners. Similarly, Barry and McCarthy’s survey showed that happy couples attributed only 15–20% of their happiness to a happy sex life, while disharmonious couples attributed 50–70% of their pain to being “out of step” in their sex life. This means that the former regards sex as just one source of intimacy and happiness, while the latter often regards sex as the main source of marital problems (Johnson, 2008). As a matter of fact, a partner who is not in a harmonious relationship fails to find the root of the problem. This is especially true in couples who have a declining quality of sexual life as they age. In the study of Hite (1981b), generally speaking, the sexual interest of partners aged 60 years and over is decreasing, but the importance of affect is increasing. In the role of affectional bond, many people can still enjoy the pleasure of sexual activities. Some follow-up studies also show that in the context of long-term mutual support, elderly couples can adapt to the obstacles of sexual intercourse caused by the decline of physical function, so that sex life can continue to contribute to their satisfactory marriage life (Hinchliff and Gott, 2004; Ménard et al., 2015). Similarly, a study by Eagle (2016) shows that in a long-term stable relationship, sexuality is more important at first, but over time, emotional intimacy and secure attachment become more important.

An additional healthy characteristic of “I-Thou” mode relationships, especially those with physical or mental sexual barriers, is adaptability. There are some couples who can accept the fact that each other has sexual barriers: they can live happily together even without good sexual behavior (Scharff, 1982). In a case reported by Blechner (2006), the sexual fantasies of a couple had been not compatible for decades. In the end, the couple completely stopped having sex, but they continued to love each other and to live a relatively stable and happy life together.

In short, in intimate interactions dominated by the “I-Thou” relationship mode, both parties can often devote themselves to the current sexual activities, so sex becomes an important way for them to express their intimacy. In the words of Slavin (2002), it is a kind of innocent sexuality. However, even if there are sexual problems, the couple can also build a love connection through good communication. In this case, the role of emotional intimacy in the relationship is more prominent, and sex exists as an auxiliary.

## Sexual Relationships Dominated by the “I-It” Mode

In addition to the ideal situation in which a relationship is dominated by the “I-Thou” mode, many unsatisfactory situations also exist. As a result, sexual behavior dominated by the “I-It” mode has gradually emerged. In this kind of relationship mode, both sides often cannot regard each other as equal subjects. This kind of monological love is not “real outgoing to the other, reaching to the other, and companying with the other.” Instead, a monological man “tries to incorporate the other into himself… the withdrawal from accepting the other person in his particularity in favor of letting him exist only as one’s own experience, only as a part of oneself” (Friedman, 2002, p.89). Contrary to the ideal case, here, sex has become a stage of repeated intimacy problems, even full of deception and violence, and has become a “battlefield” on which a power struggle between the two sides plays out. This kind of power struggle is also frequently the main topic of long-term marital conflict (Kernberg, 2011a). In the words of Slavin (2002), it is less innocent sexuality. In this type of sexual relationship, partners use some interaction strategies to complete what Brandchaft calls pathological accommodation (Brandchaft et al., 2010). Here are some typical interaction strategies.

### Strategy 1: Control

In a sexual relationship in which *control* is the main theme, in one kind of situation, the other party is used to simply obtain sexual pleasure. The compliant party in this relationship has become a pure tool of catharsis, and the emotions and needs of this party have been ignored. In this case, the feelings of both sides are often empty. The dominant party often obtains a certain degree of satisfaction, whereas the compliant party is not respected and its value is devalued. Therefore, this kind of sexual behavior is harmful to the intimate relationship. Johnson (2008) called it *sealed-off sex*. For example, in one of the cases reported, Marie (the compliant party) described the relationship as follows: “I am a blow-up Barbie for him. Our sex is so empty. It takes me to the end of alone.” In contrast, Kyle (the dominant party) said: “Since all the fighting started. I stop feeling, and sex becomes mechanical. Then I see you as ‘the woman.’ It’s safer that way. At least I know how to do sex. Closeness is harder.” Similarly, in some cases described by Scharff (1982), both husband and wife regard each other as a tool to satisfy their sexual needs, and there is no emotional intimacy between them. They even said frankly, “we are together because of sex!” In this case, their marriage relationship is very fragile, even full of discontent, resentment and despair, so conflicts often occur.

According to Simpson and Gangestad (1991), the dominant party in this relationship will avoid various actions that lead to emotional blending (e.g., caressing and kissing). This pattern of sex usually occurs in people who have been betrayed by their loved ones or who have been taught not to show too much emotion. In this way, the door of emotional connection to passionate sex is closed (Johnson, 2008). In this mode, even with multiple sexual partners, the sexual enjoyment is limited, far less than the experience in synchrony sex. The reason is that the stimulation achieved through this way of sex is strong and short-lived. If they want to maintain their passion, they have to change their sexual partners or “develop” new skills to maintain excitement (Gillath and Schachner, 2006). As de Vries (2009) said, when we don’t put in feelings during sexual intercourse and only regard others as the objects of sexual desire, we deny the psychological level of others and only see their physiological functions. At this time, both sides lost their humanity and “became animals.”

In another kind of case, the physiological satisfaction brought by sex is not the focus, but the important thing is to gain psychological control over the relationship and partner. In Hite (1981b) report, many men showed that they could gain a strong sense of conquest during sexual intercourse. This kind of expression includes “A woman’s body is always a challenge… A woman’s body is a mountain to be scaled, a house to be inhabited,” “intercourse, to me, is tied in with a subsidiary feeling of power over the woman, like that of a master and slave; it means to me that I have conquered all of her resistance,” and “Intercourse means I’ve gotten the woman. I enjoy a woman’s subservience” (p.334). In this sense, sex and aggression are inextricably linked (Kernberg, 2011b). At this point, many dirty terms related to sexual behavior also reflect this meaning, such as “laying, making, fucking, screwing, turning a trick, and scoring” (Yalom, 1980, p. 383). When sex is so inextricably linked to aggression and when individuals need to experience this sense of control, they will try every means necessary to force their partners to cooperate with their own sexual desires.

In terms of specific mechanisms leading to control, the realization of sexual coercion can be divided into three categories: language, emotion and physical action. It can be direct or obscure. For instance, when a man wants to make his partner have sex with him when his partner is not willing, he can use language to influence his partner by saying, “If you satisfy me this time, I’ll buy you a necklace tomorrow.” If the partner wants to get this “reward,” she may match his sexual needs. In addition, he can also display negative emotions to complete this manipulation, such as glaring angrily or apathetically into the face of the partner without saying a word, forcing the other party to comply with his needs. In the form of physical action, he can also use the infliction of physical pain to make the latter yield to his own violence. Of course, this kind of coercion or manipulation can also be carried out in many ways at the same time, such as violence against a partner in anger, accompanied by verbal humiliation (see Table 1 for more specific examples).

**TABLE 1:** Examples of ways to achieve sexual coercion.

Types	Concrete forms
Language	• Lying: “If you satisfy me this time, I will buy you a necklace tomorrow”;
	• Criticism/humiliation/belittling: “Bitch, you are nothing but my plaything!”;
	• Ordering: “I told you to get down. Do you hear me?”;
	• Intimidation/threat: “If you don’t meet my needs, we’ll break up/divorce,” “If you dare say no, I’ll deal with you!”;
	• Rationalization: “If you love me, comply with me”;
Emotion	• Anger: To force the other party to comply with their sexual needs;
	• Apathy: To force the other party to meet their sexual need;
Action	• Physical aggression: Hitting, with or without tools;
	• Weakening of resistance: Administration of alcohol or drugs;
	• Restriction of freedom: Restraining the other person and imprisoning them in a space.

Research has demonstrated that anxiously attached men are more likely to pressure their partners to have sex, especially when their relationship or intimacy is threatened (Davis et al., 2006; Brassard et al., 2007). Some men say that a strong sense of frustration leading to unhappiness is felt when their wives refuse to have sex. As a result, they force their wives to have sex with them in ways that include some degree of violence, such as tearing off the other’s trousers (Hite, 1981b). For many, the re-enactment of sadism and the performing of rough sex is meant to correct and improve the defective object relations: in this process, the victim unconsciously plays the role of a past perpetrator and becomes his weak “stand in.” In these situations, sexual pleasure is often a secondary goal (Juni, 2009). From this point, we can see that some criminals rape women not for sexual satisfaction, but to get away from and overcome feelings of rejection. For example, a sex offender described as follows:

*I have raped women (four rapes with one attempt) for two major reasons: (1) to gain a feeling of absolute control over a woman I felt rejected me, and (2) to prove I was as worthless as my inner turmoil made me believe I was. When I raped, I felt a commitment to finish intercourse after initiating my approach, though I didn’t want sex (I was after that feeling of utter control and domination over someone else)* (Hite, 1981b, pp. 723–724).

Further studies also show that some sex offenders have been troubled by negative emotions such as insecurity, loneliness and pain for a long time, so they hope to gain a sense of control in the process of sexual assault (Ward et al., 1996; Craissati et al., 2002; Marsa et al., 2004).

### Strategy 2: Compliance or Submission

For some, using compliance or submission strategies in sexual relations is a way of adapting; without these coping mechanisms, they will face great difficulties. One common example of such a difficulty is limitation by economic conditions. In the report of Hite (1981a), some women are forced to obey the other in sexual relations because of their financial disadvantages. In other words, they fear that if they do not meet their partner’s sexual needs, they will be subject to “economic sanctions.” For example, a married woman described her situation as follows: “I see a lot of marriages held together not with a genuine desire to share a life, but with a need to keep things financially secure” (p. 437). Another wife expressed this: “I really felt I was earning my room and board in bed for years… Now that I am self-supporting, I don’t need to play that game anymore. What a relief!” (p. 438). Although the status of women has improved greatly, this situation still exists in some intimate relationships. A more extreme example is prostitutes, who are paid financially for their sexual services. In addition, some men are compliant sexually to wealthy women because of their own economic needs. In this case, the economically disadvantaged party is often controlled by the other party and endures the other party’s different types and degrees of particularities. Therefore, in such an unequal sexual relationship, intimacy and psychological well-being are often low.

Another kind of situation that involves compliance is the comforting of the partner and cooperation in order to relieve psychological anxiety. In the *solace sex* described by Johnson (2008), one partner achieves communication and love through sexual behavior, while the other obtains a sense of attachment, which relieves negative emotions (e.g., fear and anxiety). Therefore, this type of sex has more emotional input, which can maintain the stability of the love relationship to a certain extent. In other words, the main background of this sexual behavior pattern is anxiety, and sex itself is just a way of expression: an appendage of love. However, if this pattern is obsessively repeated, the two sides will gradually enter a relationship of “excessive performance to please the other” and “endless demand,” and sexual behavior becomes a means to compensate for the lack of attachment and to ameliorate fear, leading to emotional alienation between the two partners. A client named Mandy described it as follows: “Sex with Frank is okay. But to be truthful, it’s the cuddling I really want. And the reassurance. It’s like sex is a test, and if he desires me, then I feel safe. Of course, if he ever isn’t horny, then I take it real personally and get scared.”

This type of compliance can be seen in many ambivalent attachment individuals: ambivalent individuals are usually troubled by the fear of being abandoned, so the repeatedly exhibit eagerness to satisfy the safety and love needs of their partners. Compared with the pleasure of sexual intercourse, they are more eager to get the love of the other (e.g., hugging, kissing and caring behavior). Because of the fear of abandonment, they tend to restrain their sexual needs, follow their partner’s preferences and try to please their partner (Davis et al., 2006; Birnbaum, 2010). As a result, they are also vulnerable to sexual coercion from their partners (Karantzas et al., 2016). In other words, when a partner explicitly or secretly puts pressure on them, they tend to comply and to meet their partner’s sexual needs.

In more serious cases, compliance will tend toward the state of masochism. For those who tend to be abused, their autonomy has been greatly limited and impaired, and they must be in a “symbiotic state” with their partners to obtain a sense of security (Fromm, 1956; Yalom, 1980). As a result, they are also weak and passive with regard to sexual behavior. In the face of sexual coercion of some masochists, victims usually “cooperate with the script” and comply with the other to avoid physical and psychological attacks (Juni, 2009). Some abused individuals even “like” this feeling of being forced and sexually assaulted, because it is a “proof of their love.”

### Strategy 3: Avoidance

When using avoidance strategies, individuals often choose to cut off connection with their partners, showing indifference, independence and self-sufficiency in sexual behavior (Alperin, 2001). These individuals are often resistant to and afraid of intimacy, so it is difficult for them to get a satisfactory sex life in close contact with their partners. As a result, some people choose to indulge in pornographic works (e.g., pornographic films, pictures and novels) (Yoder et al., 2005; Willock, 2013; Wood, 2021). There is no doubt that pornography can bring people strong stimulation and pleasure. However, most of the pornography is a kind of fantasy product of “sex without love,” which is often full of devaluation to women. As Hite (1981b) put it, in most pornography, women succumb to a stronger, even hostile and violent man. Even in the more “reserved” pornographic magazines, there is no sexual intercourse in the pictures, but almost all the women’s eyes and posture present a kind of seduction to men, as if they are saying “come here and occupy me.” In other words, the woman is still in the position of being controlled by male readers and used to their fullest. Therefore, if the individual is immersed in an internal loop^1^ for a long time in a closed system, it is undoubtedly strengthening an interactive way of manipulating women.

Along with the stimulation of pornography, avoidant individuals often turn to masturbation. Many studies show that masturbation is an important way to get pleasure, and it generally occurs in many different countries and is performed by people of a variety of age and gender groups (Miller, 2015). Even in the natural world, this method is used by many mammals (de Vries, 2009), such as mice, dogs and orangutans (Hite, 1981b). As mentioned above, in many satisfactory sexual relationships, masturbation can become an aid to intimacy. For instance, when the partner is not around, masturbation can be used to appropriately satisfy their desire in complete “solitude,” and watching the partner masturbate and helping the partner masturbate can promote sexual communication and coordination between the two sides. However, the root of some compulsive masturbators is that they are unable to establish a satisfactory relationship and have to indulge in “one person’s carnival.”^2^ In Kernberg (1980) study, we can see that in some borderline personality disorder clients, their sexual desire points to the self, completely excluding the possibility of others providing sexual arousal and satisfaction. Recent studies have also shown that avoidant attachment style individuals tend to deny or limit their desire for a partner. They usually obtain sexual pleasure by means of self-reliance, such as pornography and masturbation, rather than choosing to have intimate contact with their partners (Mark et al., 2018). In fact, in masturbation, the individual needs to establish a relationship with others *via* fantasy to achieve sexual pleasure (and orgasm). In other words, this kind of individual is immersed in the internal loop instead of in the needs of the external others.

In addition, even if some individuals who use avoidance strategies can have sex with their partners, they are often unable to devote themselves completely to the act. For example, when some avoidant attachment individuals have sex with their partners, they will experience a relatively strong sense of alienation and estrangement, while showing a lower level of physical emotion. Even in sexual fantasies, they show alienation from their partners (Birnbaum, 2010). For them, having sex is more a way of pretending to be intimate, with the aim of avoiding responsibility and commitment to the relationship, preventing emotional intimacy and seriously dealing with the relationship (Karantzas et al., 2016). In other words, these actions are actually avoiding sexual intercourse (and intimacy). For example, some clients with obsessive-compulsive disorder will get satisfaction again through masturbation as much as possible after having a satisfying sexual experience with others (Mitchell, 1988) so as to recover “autonomy” and declare their independence. It is not difficult to see that this kind of “one person’s sexual behavior” or “one person’s sexual satisfaction” seems to express: “I don’t need you, I can get satisfaction only by myself.” In fact, such clients are afraid of the need for others. According to Mitchell (1988), the purpose of this kind of forced masturbation is to eliminate the vulnerability and anxiety caused by interpersonal relationships. Similarly, some borderline personality disorder clients may choose to obtain sexual satisfaction with others. But after the act, they may feel degraded or even worthless (Kernberg, 1980). In fact, this “sense of depravity” is a way to belittle the importance of others, thus gaining control of others in the fantasy.

Another way to fight a partner through sex is to show a certain degree of “split.” Specifically, on the one hand, these individuals show impotence or apathy in the face of their legal partner (“relationship one”), and on the other hand, they have sexual reactions with people other than their partner (“relationship two”) (Mitchell, 1988). For example, in the case of Mitchell (2002), a client named George felt that he could not bear the control of his wife, so he paid for a prostitute’s sexual services and let the prostitute control him. Similarly, in a case reported by Kernberg (2011a), a male client had almost no sex life with his wife, but often patronized a series of high-level prostitutes, and experienced totally pleasant sexual satisfaction together without any emotional involvement with the latter. In this issue, it can be seen that among both men and women, fearful-avoidant attachment individuals tend to have more sexual partners (Favez and Tissot, 2019). After all, this is still the result of not being able to establish a satisfactory relationship with a partner, so it is through someone other than the partner that some kind of “substitution” and “compensation” is carried out. However, as described in sealed-off sex, the passion in this mode is doomed to be short and empty, because it is not completed in cooperation with a real other.

### Supplementary Notes

In the “I-It” relationship dominated sexual mode, we distinguish three typical interactive strategies: control, compliance and avoidance. In these types of interaction, either sex becomes the purpose, turning people into a tool to vent their desires; or sex becomes a means where the purpose is to achieve economic guarantee or a sense of security and control. In these cases, a relatively independent subject does not exist: either the boundary is cleared and blurred, and others become my vassals (control strategy) or I become others’ vassals (compliance strategy); or the relatedness is cut off, resulting in loneliness or “false independence” (avoidance strategy). These sexual behaviors are in sharp contrast to what Winnicott (1965) called “solitude after intercourse.” In other words, the dialectical tension between relatedness and individuation is destroyed.

It is worth noting that the interaction strategy in sexual relations is not invariable, but will change in the specific relational matrix. On this point, we can draw an analogy with Sroufe’s point of view. As described by Karen (1998), Sroufe identified three types of avoidant children: (1) “the lying bully who blames others”; (2) “the shy, spacey loner who seems emotionally flat”; and (3) “the obviously disturbed child, with repetitive twitches and tics who daydreams and shows little interest in his environment” (p. 186). It can be considered that the first type achieves interaction by manipulating others, which can only be achieved when one’s own strength is significantly stronger than that of the other; the second type is obviously afraid of interaction and continues to cut off the connection with others; the third type attempts to “meet” the needs of others by immersing themselves in the internal loop and a closed system. Similarly, as can be seen in the above descriptions, individuals who mainly use avoidance strategies may manipulate others in their fantasies and establish sexual relations in their internal loops, or have sexual relations with people other than their partners when they are stronger than the other (control strategies). Therefore, there is a significant relationship between avoidant attachment and sexual coercion (Karantzas et al., 2016). In the same way, sadism and masochism may switch roles under different power contrast, i.e., the original abuser plays the role of the victim (control strategy becomes compliance strategy), and the original victim plays the role of the abuser (compliance strategy becomes control strategy) (Mitchell, 1988).

In addition, according to Buber’s point of view, the “I-It” relationship and the “I-Thou” relationship are both necessary parts of human survival, and they complement each other. On the one hand, living in the world, we must rely on others to provide me with materials, maintain basic needs, in order to make a living. At this point, relying on and using others is indispensable. On the other hand, people tend to transcend themselves, yearn for holiness and pursue deeper meaning, which can only be achieved through the “I-Thou” relationship. Therefore, human beings live in the dual world of “I-It” and “I-Thou” relationships (Buber, 1970). In this sense, synchrony sex also needs the foundation of an “I-It” relationship; that is, “using” the body and emotion of the other party to obtain sexual satisfaction and psychological pleasure. But there is no doubt that sexual behavior dominated by “I-Thou” relationship is a healthier mode, which is more conducive to the long-term maintenance of an intimate relationship.

## Conclusion

On the basis of Buber’s intersubjective theory, this paper discusses the role of sex in intimate relationships by reference to research in psychoanalysis and attachment theory. Under the mode of “I-Thou” relationships, the subjectivity of the partner can be fully demonstrated: both parties can often fully participate in the current sexual behavior and respond wholeheartedly. In this process, sex and love achieve a healthy combination. When there incoordination (or even conflict) arises in sexual activities, both parties can negotiate with each other sincerely and even repair the relationship after rupture. At this time, compared with sex, the role of affect is more prominent. In the mode of “I-It” relationships, sex acts more as a tool to achieve a certain purpose (e.g., economic guarantee, sense of security and sense of control), so the intersubjective tie of partners is abnormal: either the boundary is cleared and blurred, others become my vassal (control strategy), or I become others’ vassal (compliance strategy); or the bone is cut off, leading to loneliness or “false independence” (avoidance strategy). It is not hard to see that intimacy dominated by “I-Thou” relationship is more adaptable. Thus, the guiding of lovers or couples who are trapped in the “I-It” relationship to the “I-Thou” relationship remains the goal of further work.

## Data Availability Statement

The original contributions presented in the study are included in the article/supplementary material, further inquiries can be directed to the corresponding author/s.

## Author Contributions

The author confirms being the sole contributor of this work and has approved it for publication.

## Conflict of Interest

The author declares that the research was conducted in the absence of any commercial or financial relationships that could be construed as a potential conflict of interest.

## Publisher’s Note

All claims expressed in this article are solely those of the authors and do not necessarily represent those of their affiliated organizations, or those of the publisher, the editors and the reviewers. Any product that may be evaluated in this article, or claim that may be made by its manufacturer, is not guaranteed or endorsed by the publisher.

## Footnotes

1 In this paper, the internal loop refers to the interaction between the individual and others in the internal world, and its meaning is similar to internal object relationship, the “expectation” of interaction described by Beebe and Lachmann (2002), “implicit relationship knowing” described by the Boston Change Process Study Group (BCPSG, 2010), and the “internal working models” in attachment theory (Wallin, 2007).

2 In this sense, fetishism is also an avoidance strategy. Fetishists cannot automatically relate to a part of a woman or accessories (e.g., underwear, shoes, or even a woman’s feet). In this way, they can experience strong sexual impulses and pleasure. However, they are unable to establish a relationship with the other as a whole (Yalom, 1980).
